# Survival of patients with multidrug-resistant tuberculosis in Central China: a retrospective cohort study

**DOI:** 10.1017/S0950268820000485

**Published:** 2020-02-19

**Authors:** Jianjie Wang, Meilan Zhou, Zi Chen, Cong Chen, Gang Wu, Yingping Zuo, Xin Ren, Zhuan Chen, Weihua Wang, Yu Pang

**Affiliations:** 1 Department of Tuberculosis Control, Wuhan Pulmonary Hospital, Wuhan, China; 2 National Clinical Laboratory on Tuberculosis, Beijing Key Laboratory on Drug-resistant Tuberculosis Research, Beijing Chest Hospital, Capital Medical University, Beijing Tuberculosis and Thoracic Tumor Institute, Beijing, China

**Keywords:** Multidrug-resistant, risk factor, survival, tuberculosis

## Abstract

The aim of this study was to evaluate long-term survival and risk factors associated with multidrug-resistant tuberculosis (MDR-TB) patient survival in Central China. Between December 2006 and June 2011, incident and retreatment adult MDR-TB patients were enrolled in the present study. Cox proportional hazard regression analysis was used to evaluate the risk factors affecting survival. The total follow-up period was 270 person-years (PY) for 356 MDR-TB cases in Wuhan. Of the 356 cases, 103 patients died, yielding an average case fatality rate of 381.2 per 1000 TB patients per year. Using adjusted Cox regression analysis, older age (adjusted hazard ratio (aHR) >3.0 starting from 30 years) and low education level (primary and middle school; aHR 1.67 (95% CI 1.01–2.77)) were independently associated with lower survival. Diabetes mellitus profoundly affected the survival of MDR-TB patients (aHR 1.95 (95% CI 1.30–2.93)). Our data demonstrate that coexistent diabetes significantly and negatively impacted MDR-TB patient survival. In addition, MDR-TB patients aged 60 years or older exhibited a greater risk of mortality during follow-up. Our findings emphasise that MDR-TB patients with comorbidities that increase their risk of death require additional medical interventions to reduce mortality.

## Background

Multidrug-resistant tuberculosis (MDR-TB), defined as a disease caused by infection with *Mycobacterium tuberculosis* (MTB) strains with resistance to at least isoniazid and rifampin, is a major threat to global TB control efforts [1,2]. In 2017, it was estimated that of 460 000 incident MDR-TB cases, only 25% were actually diagnosed and treated [1]. Because treatment regimens for MDR-TB tend to have low effectiveness, are poorly tolerated and extremely expensive, patients with MDR-TB are more likely to experience poor outcomes and death [3,4]. In fact, a previous meta-analysis demonstrated that only half of MDR-TB patients treated with second-line regimens achieved successful clinical outcomes after treatment completion [5]. Treatment failures of MDR-TB cases not only resulted in an increased hazard ratio for death, but also accelerated the transmission of this form of TB within the community [6].

China has been described as a global ‘hotspot’ of MDR-TB, accounting for 11% of disease burden worldwide [1,7]. According to a national survey on drug-resistant TB, MDR-TB represents 5.7% of new cases and 25.6% of previously treated cases in China [7]. The high rate of MDR-TB in this country continues to reverse recent strides made in recent decades toward reducing TB morbidity and mortality. More importantly, despite the serious threat posed by MDR-TB, insufficient attention has been paid to diagnose and treat MDR-TB patients [7]. In China, a policy of free drug treatment for TB covers the costs of an entire course of first-line anti-TB drugs, while the extremely expensive second-line anti-TB drugs for MDR-TB treatment must be paid for by health insurance and patient payments of out-of-pocket costs [8,9, ]. Therefore, a sizeable proportion of MDR-TB patients incur catastrophic financial hardship stemming from the high costs of TB medical care that they frequently cannot afford, resulting in unplanned treatment interruption [10]. This situation has undoubtedly contributed to the increasing mortality and transmission dynamics of MDR-TB epidemics in China. Consequently, several studies have focused on factors contributing to the high case fatality rate of the MDR-TB patient cohort [2,11], but data on patient survival and factors influencing the length of survival are limited, especially in settings with high prevalence rates of MDR-TB.

To address this issue, here a retrospective study was carried out among all MDR-TB patients in Wuhan, the largest city in Central China. We analysed the survival of a cohort of MDR-TB patients over a 10-year period (2006–2016). Our aim was to evaluate long-term survival and risk factors associated with the reduced survival of MDR-TB patients.

## Methods

### Setting and study population

Wuhan, the capital of Hubei Province, has an area of 8494 km^2^ and a population of 10.91 million. In 2010, Wuhan had a TB case notification rate of 436.3 per 100 000 people per year, with 6.9% of new cases and 18.8% of previously treated cases diagnosed with MDR-TB [12]. This retrospective 10-year study was conducted in Wuhan Pulmonary Hospital, the hospital supervising TB control activities in Wuhan. Both new (incident) and retreatment MDR-TB adult patients were enrolled in the present study after diagnosis using conventional *in vitro* drug susceptibility testing (DST). All patients received follow-up care for 5–10 years after diagnosis. Demographic and clinical data were extracted from the National Tuberculosis Registry database and from patient medical records held at Wuhan Pulmonary Hospital. Follow-up data on the survival and cause of death were obtained by interviewing patients or their relatives via telephone once a year. Briefly, the patients were first interviewed via telephone, and those who could not be found by telephone calls were interviewed with their relatives via telephone. The censored categories refer to: (i) loss to follow-up; (ii) survival by the end of follow-up period; (iii) death from a competing risk.

### Laboratory examination

Sputum specimens were submitted to the laboratory for mycobacterial culture as previously described [13]. Bacterial colonies were harvested 4–8 weeks and subjected to conventional *in vitro* DST and MTB species identification testing [13]. The drugs tested in these studies included isoniazid, rifampin, ethambutol, streptomycin, kanamycin and ofloxacin. Pre-extensively drug-resistant tuberculosis (pre-XDR-TB) was defined as a disease caused by MDR-TB isolates with additional resistance to either one fluoroquinolone (FQ) or one second-line injectable drug (SLID) but not both. XDR-TB was caused by MDR-TB isolates that were additionally resistant to one FQ and one SLID.

### Treatment

From December 2006 to June 2011, MDR-TB patients received a standardised antibiotic treatment regimen previously developed by the Global Fund. The abbreviation of each drug was as follows: Am, amikacin; Cm, capreomycin; Cs, cycloserine; E, ethambutol; Lfx, levofloxacin; Mfx, moxifloxacin; PAS, *p*-aminosalicylic acid; Pto, protionamide; Z, pyrazinamide. Treatment consisted of a 6-month intensive phase followed by an 18-month continuation phase of: 6 Z, Am (Cm), Lfx (Mfx), Cs (PAS, E), Pto/18 Z, Lfx (Mfx), Cs (PAS, E), Pto. Treatment outcomes were categorised according to the standard defined WHO terms that included cure, complete, death during treatment, failure, loss to follow-up and not evaluated [14,15]（Table 1).

**Table 1.** Treatment outcome definitions in MDR-TB patients

Outcomes	Definitions
Cure	At least 3 consecutive negative cultures and no positive culture during the last 18 months of treatment
Treatment completion	Bacteriological conversion through the end of treatment but fewer than three consecutive negative culture
Default	Treatment interruption for 2 or more consecutive months for any reason without medical approval
Treatment failure	Persistence of two or more positive cultures of the five cultures noted in the final 12 months, persistence of one or more positive cultures of the final 3 months, or early treatment termination due to poor clinical or radiological response or adverse events
Death	Death for any reason during the course of MDR-TB treatment

### Statistical analysis

Double entry of data was conducted using the EpiData Software Package (EpiData Assoc., Odense, Denmark). Death was defined as mortality from TB during anti-TB treatment or follow-up periods. The *χ*^2^ analysis of variance tests were used to compare categorical variables. Case fatality rates (per 100 person-years (PY)) and Kaplan–Meier survival curves were analysed to estimate survival rates. We used crude and adjusted Cox proportional hazard regression analysis to identify and evaluate the factors impacting survival, including demographic characteristics, initial drug susceptibility profiles and clinical treatment. Death from MDR-TB was designated as follow-up termination. In addition, variables were selected using a forward LR method and remained in the model for a *P* value of <0.1. The adjusted hazard ratio (aHR) was calculated to describe the relative hazards affecting MDR-TB patient survival. SPSS software version 20.0 (SPSS Inc., Chicago, IL) was used for all analyses. A *P* value of <0.05 was considered statistically significant.

## Results

### Demographics

A total of 356 MDR-TB cases reported in Wuhan during 2006–2011 were included in this study. Cases included 262 male and 94 female patients that together had an overall mean age (range) of 46.45 (16–78) years. Of these study subjects, 165 (46.3%) were unemployed. A previous history of TB disease was reported by 302 patients. Previously treated MDR-TB cases had undergone a mean of 1.83 failed courses of treatment. The most frequent comorbidity in this study was diabetes (46/103, 44.7%), followed by cirrhosis (6/103, 5.8%) and kidney disease (2/103, 1.9%). HIV tests were conducted on all patients and no patients were found to be seropositive. Patients were infected with organisms that were resistant to a mean of 3.48 (2–6) drugs, with 104 and 12 patients diagnosed with pre-XDR-TB and XDR-TB, respectively (Table 2).

**Table 2.** Mortality of multidrug-resistant tuberculosis cases stratified to demographic and clinical characteristics

Characteristics	Total			Mortality		
	*n*	*N*	%	Person-years	Deaths	Rate; 95% CI
Gender						
Female	94	356	26.4	60	22	36.41 (23.97–55.3)
Male	262	356	73.6	210	81	38.62 (31.06–48.02)
Age group (years)						
<30	58	356	16.3	6	4	65.75 (24.68–175.19)
30–60	233	356	65.4	182	69	37.96 (29.98–48.06)
>60	65	356	18.3	82	30	36.44 (25.48–52.12)
Residence						
Urban	238	356	66.8	189	69	36.43 (28.77–46.12)
Rural	118	356	33.2	81	34	42.11 (30.09–58.93)
Education level						
High school and university	137	356	38.5	62	21	33.83 (22.06–51.89)
Primary and middle school	219	356	61.5	208	82	39.41 (31.74–48.93)
Marriage status						
Unmarried	29	356	8.2	41	10	24.14 (12.99–44.87)
Married	327	356	91.8	229	93	40.66 (33.18–49.82)
Occupation						
Worker	78	356	21.9	36	14	39.34 (23.3–66.43)
Farmer	113	356	31.7	81	34	42.11 (30.09–58.93)
Retired	58	356	16.3	65	25	38.22 (25.83–56.56)
Unemployed	107	356	30.1	88	30	33.93 (23.72–48.53)
Treatment history						
New cases	54	356	15.2	3	1	32.43 (4.57–230.23)
Retreated cases	302	356	84.8	267	102	38.19 (31.45–46.37)
Diabetes						
No	263	356	73.9	157	57	36.21(27.93–46.94)
Yes	93	356	26.1	113	46	40.80 (30.56–54.47)
Cirrhosis						
No	329	356	92.4	256	97	37.89 (31.05–46.23)
Yes	27	356	7.6	14	6	42.86 (19.25–95.4)
Kidney disease						
No	348	356	97.8	265	101	38.11 (31.36–46.32)
Yes	8	356	2.2	5	2	40.0 (10–159.94)
Initiation of treatment						
Yes	231	356	64.9	128	39	30.47 (22.26–41.7)
No	125	356	35.1	142	64	45.07 (29.71–48.5)
Drug resistance pattern^a^						
MDR	252	356	70.8	175	64	36.59 (28.64–46.75)
pre-XDR/XDR	104	356	29.2	95	39	40.94 (29.91–56.03)

a MDR, multidrug-resistance, no additional resistance to FQs or second-line injectable drugs; XDR, extensive drug-resistance; pre-XDR, MDR plus additional resistance to either any FQ or any second-line injectable drug but not both.

Of 356 MDR-TB cases, 231 (64.9%) received treatment with second-line drug treatment regimens, while the remaining 125 (35.1%) refused treatment in the Wuhan Pulmonary Hospital. Among 231 cases undergoing treatment, 167 (72.3%) were cured, two (0.9%) died, 31 (13.4%) defaulted and 31 (13.4%) failed treatment. Of the 31 patients who had defaulted treatment, the average treatment period was 7.8 months (95% CI 5.5–10.2 months).

### Survival analysis

The total follow-up period was 270 PY，the mean follow-up time was 2.3 (1.25–3.67) PY. Of the entire patient sample, 103 patients died, yielding an average case fatality rate of 381.2 per 1000 TB patients per year (95% CI 314.25–462.4). Case fatality rates were highest among patients with diabetes, those in the rural population and patients infected with pre-XDR/XDR, whereas the lowest case fatality rates were observed among unmarried patients and new TB cases.

**Fig. 1.**
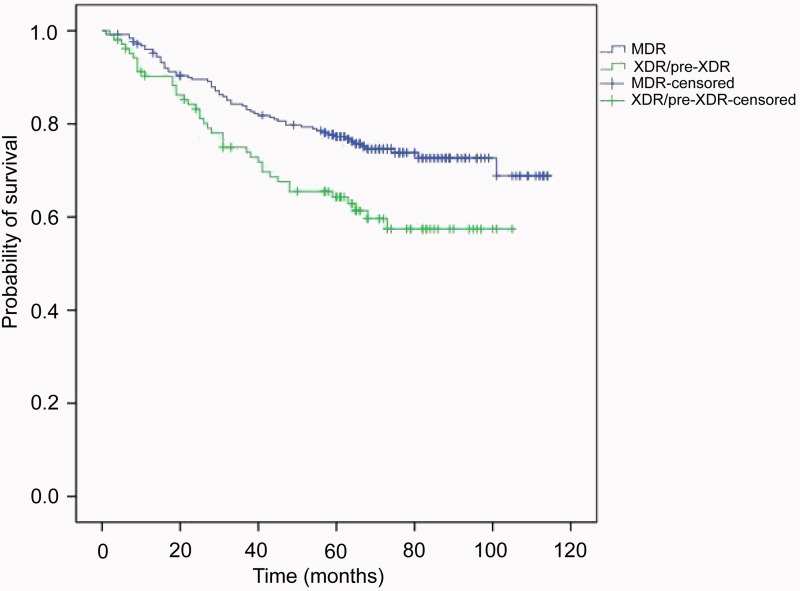
Kaplan–Meier overall survival curve of 356 MDR-TB patients according to the drug-resistant pattern.

Using adjusted Cox regression analysis, older age (aHR >3.0 starting from 30 years) and low education level (primary and middle school; aHR 1.67 (95% CI 1.01–2.77)) were independent factors associated with lower survival. Previous treatment history profoundly affected the survival of MDR-TB patients (aHR 13.15 (95% CI 1.82–94.96)) (Fig. 1). In addition, patients with diabetes mellitus were associated with a higher risk of dying during the follow-up period (aHR 1.95 (95% CI 1.30–2.93)). As a factor independent of the aforementioned factors, the patients refusing treatment were at elevated risk for mortality in our observation (aHR 2.98 (95% CI 1.95–4.54)). In contrast, adjusted analyses revealed that gender, residence, marriage status or occupation had no significant effect on survival (Table 3).

**Table 3.** Factors associated with mortality among the total MDR-TB cases

	Crude HR (95% CI)	*P* value	Adjusted HR (95% CI)	*P* value
Gender				
Female	Reference			
Male	1.42 (0.89–2.28)	0.142		
Age group (years)				
<30	Reference		Reference	
30–60	5.01 (1.83–13.74)	0.002	3.03 (1.08–8.52)	0.035
>60	9.29 (3.27–26.40)	<0.001	3.72 (1.26–11.02)	0.018
Residence				
Urban	Reference			
Rural	1.03 (0.68–1.55)	0.900		
Education level				
High school and university	Reference		Reference	
Primary and middle school	2.90 (1.80–4.69)	<0.001	1.67 (1.01–2.77)	0.044
Marriage status				
Unmarried	Reference			
Married	0.88 (0.46–1.68)	0.691		
Occupation				
Worker	Reference			
Farmer	1.85 (0.99–3.44)	0.054		
Retired	2.85 (1.48–5.49)	0.002		
Unemployed	1.63 (0.86–3.07)	0.134		
Treatment history				
New cases	Reference		Reference	
Retreated cases	20.81 (2.90–149.23)	0.003	13.15 (1.82–94.96)	0.011
Diabetes				
No	Reference		Reference	
Yes	3.07 (2.08–4.53)	<0.001	1.95 (1.30–2.93)	0.001
Cirrhosis				
No	Reference			
Yes	0.71(0.31–1.62)	0.413		
Kidney disease				
No	Reference			
Yes	0.82 (0.20–3.12)	0.778		
Initiation of treatment				
Yes	Reference		Reference	
No	4.46 (2.99–6.65)	<0.001	2.98 (1.95–4.54)	<0.001
Drug resistance pattern^a^				
MDR	Reference		Reference	
XDR/pre-XDR	1.75 (1.17–2.60)	0.006	1.45 (0.97–2.18)	0.072

HR, hazard ratio; CI, confidence interval.

a MDR, multidrug-resistance, no additional resistance to FQs or second-line injectable drugs; XDR, extensive drug-resistance; pre-XDR, MDR plus additional resistance to either any FQ or any second-line injectable drug but not both.

## Discussion

Although TB mortality has undergone a progressive decline worldwide over the past few decades, TB remains to be one of the leading causes of death from communicable disease, while also markedly contributing to the epidemic of MDR-TB [1]. A decline in mortality from MDR-TB would not only depend on decreased death rate during anti-TB therapy, but also on the achievement of long-term survival after treatment. In this study, we first identified the factors associated with the long-term survival of MDR-TB patients in a resource-limited medical care setting. Our data demonstrate that coexisting diabetes had a significant negative impact on the survival of MDR-TB patients. However, these results do not align with the results of a recent study by Siefert and coworkers using follow-up data of patients with suspected drug-resistant TB; that study failed to show any association between survival and diabetes status [16]. This discrepancy in results may be due to a small sample size stemming from the low diabetes prevalence among patients in that study [16]. Meanwhile, a series of other reports also have demonstrated that diabetes confers an increased risk of treatment failure and death compared with the corresponding risk for non-diabetic patients [17]. Moreover, TB patients with diabetes also appear to have a high risk of relapse, even after achieving favourable outcomes [18,19]. Thus, when taken together, high rates of treatment failure and post-treatment relapse appear to result in an overall high case fatality rate for diabetic TB patients. We speculate that diabetic status, associated with hyperglycaemia and poor glucose control, leads to a dysfunctional immune response to MTB in diabetic patients, which may explain much of the association between diabetes and increased risk of death [19]. Consequently, our results highlight that optimised glucose control should be an essential component of diabetic patient treatment management to maximise the chances of prolonged survival (Table 1).

It is notable that MDR-TB patients aged 60 years or older had a greater risk of mortality during follow-up. In fact, several experts have suggested that high TB mortality in the elderly may be associated with weakened immunity and a greater number of comorbidities [20,21]. In our cohort, this association continued to be observed even after we adjusted for additional comorbidities. This finding suggests that older MDR-TB patients experienced a higher likelihood of death due to waning immunity associated with advanced age. Another potential explanation for the high case fatality rate in the elderly is that they may, as a group, experience particularly delayed diagnosis and treatment. Moreover, numerous researchers have revealed that elderly TB patients tend to exhibit non-specific disease presentation at TB onset, such as negative bacterial detection results and ambiguous clinical findings, which could delay diagnosis [22,23]. Although AFB smear testing is the standard laboratory method used for TB screening, more effective diagnostic methods with improved sensitivity are needed for the testing of paucibacillary sputum samples collected from elderly patients to achieve earlier detection of MTB. Earlier detection would likely minimise interventional delays to improve elderly patient treatment outcomes.

Another important finding of this study was that more than one-third of MDR-TB patients refused anti-TB treatment. Although the exact reasons underlying patient refusal of treatment remain unclear, lack of treatment would undermine control measures to reduce TB prevalence within the community, while also preventing mortality in these cases. Furthermore, untreated MDR-TB patients would remain a reservoir for TB transmission by supporting bacterial propagation that would likely accelerate the transmission of MDR-TB to the community. Therefore, to ultimately control TB in the community, more effective strategies are needed to address this public health concern at the level of the individual patient in China.

Our study had several obvious limitations. First, because *in vitro* second-line DST results for only KAN and OFLX were obtained in this study, incomplete DST results made it impossible to assess the impact of cross-resistance to FQs and SLIDs on treatment outcomes. Second, despite observations that additional drug resistance is an independent risk factor associated with the mortality of MDR-TB patients [24], low prevalence of pre-XDR- and XDR-TB in our cohort reduced our ability to evaluate the correlation between different drug resistance profiles and long-term survival. Third, since this study was conducted at only a single site, our findings should therefore be verified using larger epidemiological and clinical studies of patients from geographically diverse sites. Fourth, although this study demonstrated an association between diabetes comorbidity and decreased survival, diabetic severity was not investigated for its impact on long-term survival. Finally, it is an interesting topic to assess a difference in case fatality rates during and after treatment, whereas only three patients died during the treatment period. Hence, the small sample size was insufficiently large for statistical analysis. Despite these limitations, this study provides new insights to increase the understanding of risk factors contributing to the mortality of patients living in an area of high TB prevalence. Such studies are essential for formulating appropriate strategies to improve high-risk patient outcomes.

## Conclusion

In this study, we demonstrated that coexistent diabetes had a significant negative impact on the survival of MDR-TB patients. Moreover, the presence of XDR- and pre-XDR-TB disease, irrespective of FQ- or SLID-resistance, led to poorer long-term survival compared with the survival of MDR-TB patients. In addition, MDR-TB patients aged 60 years or older had a greater risk of mortality during follow-up. Our findings suggest that MDR-TB patients with certain comorbidities should receive treatment interventions aimed at reducing their particularly high risk of mortality.
